# Estimating Exerted Hand Force via Force Myography to Interact with a Biaxial Stage in Real-Time by Learning Human Intentions: A Preliminary Investigation

**DOI:** 10.3390/s20072104

**Published:** 2020-04-08

**Authors:** Umme Zakia, Carlo Menon

**Affiliations:** Menrva Research Group, School of Mechatronic Systems and Engineering Science, Simon Fraser University, Metro Vancouver, BC V3T 0A3, Canada; uzakia@sfu.ca

**Keywords:** force myography signal, exerted hand force, intended arm motion, biaxial stage, planar workspace, collaborative interactions, machine learning

## Abstract

Force myography (FMG) signals can read volumetric changes of muscle movements, while a human participant interacts with the environment. For collaborative activities, FMG signals could potentially provide a viable solution to controlling manipulators. In this paper, a novel method to interact with a two-degree-of-freedom (DoF) system consisting of two perpendicular linear stages using FMG is investigated. The method consists in estimating exerted hand forces in dynamic arm motions of a participant using FMG signals to provide velocity commands to the biaxial stage during interactions. Five different arm motion patterns with increasing complexities, i.e., “x-direction”, “y-direction”, “diagonal”, “square”, and “diamond”, were considered as human intentions to manipulate the stage within its planar workspace. FMG-based force estimation was implemented and evaluated with a support vector regressor (SVR) and a kernel ridge regressor (KRR). Real-time assessments, where 10 healthy participants were asked to interact with the biaxial stage by exerted hand forces in the five intended arm motions mentioned above, were conducted. Both the SVR and the KRR obtained higher estimation accuracies of 90–94% during interactions with simple arm motions (x-direction and y-direction), while for complex arm motions (diagonal, square, and diamond) the notable accuracies of 82–89% supported the viability of the FMG-based interactive control.

## 1. Introduction

Human robot interaction (HRI) is observed as a growing interest in manufacturing environments. In recent years, robots working in collaboration with human workers in final assembly lines have boosted up productivity [1,2,3]. A variety of measures, such as vision systems (cameras, image/depth sensors, and tracking systems), ultrasonic or wideband/radio frequency (RF) transceivers, proximity detections (capacitive, inductive, infrared, or magnetic sensors) are implemented for surveying, monitoring and sharing the workplace [4,5,6]. These tools can be either attached to the robot or installed in the workspace. However, proper supervision is hard due to obstructions of signals, limited signal ranges, and difficulties of installations within workspace. Besides, a dynamic and unpredictable environment during collaboration introduces uncertainties increasing risks of injuries for workers [7,8,9]. To address such variabilities, some input signals from human would help implementing better collaboration. Therefore, current researches focus on including biosignals for understanding human activities during interactions with machines [10,11]. Utilizing wearable sensing devices based on biosignals provides more flexibility in movements and insight about human intentions. Among such biosignals, the traditional surface electromyography (sEMG) has been studied for the last few decades. This is a noninvasive technique that can interpret electrical activities associated with muscle contraction variations under the skin. This typical technique has been studied for gesture recognition, hand force recognition, prosthetic limb controls, and rehabilitation applications [12,13,14,15].

Recently, an innovative wearable sensing system integrating the noninvasive force myography (FMG) technique has gained interest. It can be a viable solution to force measurements of upper/lower extremity by assessing muscle voluntary contractions using force-sensing resistors (FSRs), of which the resistances change with applied forces on their surfaces [16]. FSRs are small, easy to use, low-cost and repeatable, which is essential for any wearable sensor. As a contemporary technique to the sEMG, FMG signals has proven their potential in similar researches of finger and gesture recognition, isometric grasp/hand force recognition, prosthetic upper limb control, rehabilitation applications, and assistive solutions to activities in daily life (ADLs) of elders [17,18,19,20,21,22]. Surprisingly, this technique is found comparable and sometimes better than the sEMG in those applications. Recent researches have shown the FMG technique is advantageous over the sEMG in terms of lower cost, simple signal processing, and easy incorporation as a wearable device and is a better choice for human–machine interfacing than the sEMG [23,24,25].

In physical HRIs (pHRIs), interactions between humans and robots mostly occur through hand activities such as object handling or transportation tasks in simple and fixed trajectories. These require dynamic arm movements, while force interactions occur with hands. Learning human intentions such as realizing human hand forces and arm motions have been studied extensively to facilitate these collaborative tasks [26,27,28,29]. Such hand forces can be estimated by utilizing sEMG signals and isometric hand forces with FMG signals. Although the sEMG has been researched from the last few decades to estimate hand forces or positions for improving human–robot interactions, there is a gap in research for FMG-based HRI control. Therefore, our objective was to investigate the recognition of exerted hand forces in motions using FMG signals in collaboration with a simple linear robot.

In this study, real-time isotonic hand force estimation in dynamic two-dimensional (2D) arm motions using FMG was investigated for the first time. A variety of simple and complex isotonic arm movements involving shoulder abduction/adduction and elbow flexion/extension were performed continuously within the workspace. A novel scheme to interact with a biaxial stage by estimating an exerted hand force in an intended arm motion with FMG signals was implemented. The biaxial setup of two-degree-of-freedom (2-DoF; xy-plane) motions used in this study resembled a 2-DoF robotic arm. It was a type of gantry-positioning stage sometimes known as a linear or cartesian robot. Learning human intention was defined as an exerted hand force in a motion in a desired path trajectory during interactions. In a real-time scenario, a participant wearing FMG bands grasped a custom-made gripper mounted to a stage and interacted with an exerted force in an intended motion in the XY-plane. In addition, the biaxial stage adjusted its velocity with the estimated exerted hand force, so that the gripper would slide accordingly in the same trajectory, thus ensuring compliant collaboration. Two well-established supervised machine learning techniques, the support vector regressor (SVR) and the kernel ridge regressor (KRR) with their selected features, were implemented for force estimation from FMG readings, and their performances were observed separately.

Recognizing different arm motion patterns in two dimensions with real-time FMG signals was interesting and challenging because of the dynamic nature of motions and individual-specific muscle contractions. As a preliminary study, a simple interactive setup with a constrained protocol allowed investigating force estimations in a variety of complex motions with FMG signals. These motions were examined for understanding human intentions of manipulating a stage in any direction. The admittance control strategy implemented in this investigation utilized supervised machine learning methods. In many interactive control systems, complex human arm dynamics modeling is required [30]; while this study showed possible interactions without such modeling. In addition, FMG-based force estimation could be beneficial in implementing safe collaboration while working with a robot. If safety issues arise such as to avoid unwanted contacts or impact forces from the manipulator, participants can force the robot to move it further away in a certain direction. Therefore, using FMG biosignals as the only input in learning human activities appears promising.

The remainder of the paper is organized as follows: Section 2 describes the materials and methods used for real-time FMG-based integrated control, the experimental setup, and the protocol followed during different phases of real-time control. The obtained results are presented and analyzed in Section 3 and are discussed in Section 4. The paper concludes with final remarks and future work in Section 5.

## 2. Materials and Methods

### 2.1. Experimental Setup

#### 2.1.1. FMG Bands

**Figure 1:**
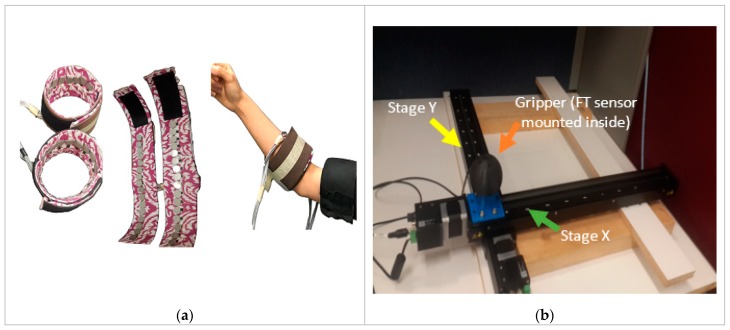
(**a**) Two customized force myography (FMG) bands worn on the upper-extremity of a participant to read muscle contraction; (**b**) a biaxial stage with a knob-like gripper mounted on its top.

Two custom-made wearable FMG bands were used to track muscles volumetric changes from the upper extremity of human participants during specific arm motions, as shown in Figure 1a. The bands were made of FSRs, of which the resistances changed as the muscles contracted. The FSRs exhibited high resistances (~10 MΩ) at no pressure, and their resistances decreased as the pressure increased (during interactions). Each band had 16 FSRs (TPE 502C, Tangio Printed Electronics, Vancouver, Canada) [31] and was approximately 30 cm long. Using voltage dividers to extract signals from these sensors, two data acquisition (DAQ) devices with a 16-channel analog input (NI USB 6259 and 6341, National Instruments, Austin, TX, US)) were used for FMG data acquisition from these bands at 50 Hz.

#### 2.1.2. The Biaxial Stage 

This 2-DoF linear robot consisted of two linear stages (X-LSQ450B, Zaber Technologies, Vancouver, Canada) with built-in motor controllers for desired translational movements [32]. Figure 1b shows the biaxial stage with a gripper. The stages had a 450 mm travel distance and supported up to a 1 m/s speed and a 100 N thrust; they were chosen because of high stiffness and back drivability. A binary communication protocol was used to exchange commands between the biaxial stage and the FMG-based real-time integrated controller at 9.6 kbps. A customized three-dimensionally (3D) printed knob as a gripper using the ABS (acrylonitrile butadiene styrene) material was mounted on top of the biaxial stage; this allowed participants to grab the gripper/knob and applied forces to slide it on a planar surface during real-time interactions. External forces acting upon the biaxial stage were measured using a 6-axis force torque sensor (mini45, ATI industrial automation, Apex, NC, USA) [33]. It was placed inside the gripper/knob to measure the true label data of exerted hand forces during interactions. NI DAQ 6210 (National Instruments, Austin, TX, USA) was used for data acquisition from the sensor. The workspace of the biaxial stage (450 mm × 450 mm) was within the maximum working area for a human operator (average size: 683 mm × 485 mm) [34]. In addition, each stage could support up to a 20 kg load capacity, while the maximum masses of a human hand were around 1 kg for female and 1.2 kg for male to interact during hand movement [35]. The biaxial stage was positioned and secured firmly on a heavy table with wooden supports for a uniform load distribution on the entire workspace.

### 2.2. Real-Time FMG-Based Integrated Control

In this study, human robot collaboration was observed, as a participant interacted with a biaxial stage in an intended arm motion. The wearable FMG bands on the upper extremity read muscles contraction during interactions. The FSRs used in the bands had an active area of 14.7 mm, actuation forces less than 15 g, and a pressure sensitivity range of 100–200 psi (pound of forces per square inches) and could easily sense muscle contractions in the underlying skin. The collaborative task was defined as the interactions between a participant to manipulate the biaxial stage by grasping its gripper to apply hand forces while moving his/her arm in an intended trajectory; the biaxial stage would adjust its velocity proportionate to the applied force, and the gripper would slide in the same path. Five different 1-degree-of-freedom (1-DoF) and 2-DoF arm trajectories were selected as intended motions. For simplicity, the collaborative task would be termed as *1-DoF/2-DoF interactions* throughout the paper. Each arm motion with a grasping force had its unique characteristics represented by multichannel FMG signals. Thus, to identify a variety of intended motion patterns, these signals could represent muscle groups active in interaction.

**Figure 2:**
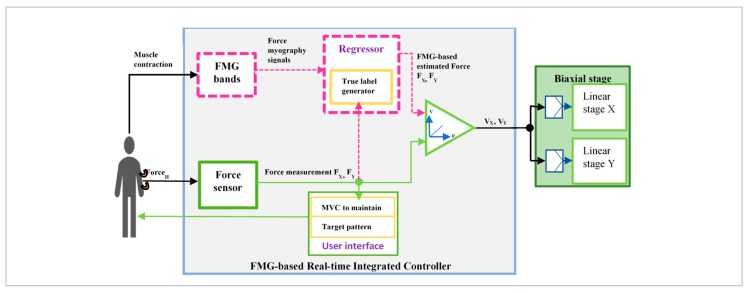
FMG-based real-time (RT) force control of a biaxial stage: the data collection and training phase are shown in green color, and the RT test phase is shown in magenta color.

An interactive control using a supervised regression algorithm estimated an applied hand force in a dynamic arm motion with FMG signals, converted it to the speed and sent the estimated data to the biaxial stage, thus allowing participants to manipulate the stage by grasping its gripper in the same trajectory. For the supervised training, force sensor (mounted inside the gripper) data was used as a true label data generator. The data acquisition and design aspect of this proposed integrated control is shown in Figure 2. During the real-time data collection for training, the biaxial stage was manipulated by the force sensor reading of the exerted hand force. During the real-time test phase, the integrated controller allowed participants to manipulate the biaxial stage with FMG-based estimated hand forces. The user interface provided a visual feedback to the participants about the target arm motion pattern and the exerted force to maintain muscle volumetric contraction (MVC) in certain ranges.

For the real-time control design, a traditional admittance control scheme was implemented [36]. Therefore, external forces applied to a gripper/knob were translated into torques at each joint, and the stage moved to a new position based on the calculated displacement. The kinematics of the considered biaxial stage was shown as:

(1) $$\left(\begin{array}{c} x\\ y \end{array}\right)=\left(\begin{array}{c} 1\;\;\;0\\ 0\;\;\;1 \end{array}\right)\;\left(\begin{array}{c} d_{x}\\ d_{y} \end{array}\right)$$

where *x* and *y* are the end positions of the gripper, and *d_x_* and *d_y_* are the displacements of the biaxial stage in the x- and y-directions, respectively. Actual and estimated hand forces (*N*) were first converted to velocities (*mm/s*) for the motor controllers of the stage and then as microsteps (*displacements*) per seconds according to Equation (4) as in the documents from Zaber technologies, which was written as [32]:

(2) Displacementx,y=Velocityx,y/(Microstepsizex,y/1000/1.638).

**Figure 3:**
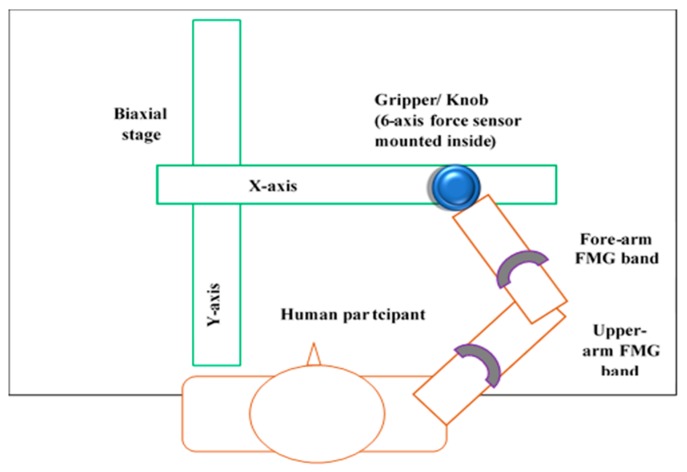
Top view of a human participant wearing FMG bands on upper-extremity (UE) interacts with the biaxial stage by grasping the gripper/ knob.

Interactions between a participant and the biaxial stage in real time are shown in Figure 3. The participant could freely slide the gripper on the entire workspace by an exerted force. All mechanical devices mentioned in Table 1 were synchronized properly for real-time operation, and fine tunings were implemented to reduce the stiffness and smooth control of the manipulator. These measurements helped the participant to reduce muscles fatigue and to interact with the biaxial stage easily while avoiding damages to the hardware.

Controlling the biaxial stage in real time with dynamic forces exerted by human hands involved careful system design considerations. The allowed external forces applied to stage were between 3 and 90 N, and certain ranges of MVC (30% and 80%) were maintained to keep the limits of the maximum thrust for proper operations of the linear stages. The smooth sliding of the gripper was ensured for the participant’s comfort and ease of control. An HP Zbook laptop (HP, Palo Alto, California, USA) with Intel Core i7 was used for implementing the real-time FMG-based integrated controller. The higher data acquisition rate (50 Hz), high-speed computations, and data transmission to the biaxial stage (9.8 kbps) with a minimal time delay (within 8 ms) made the real-time control achievable.

**Figure 4:**
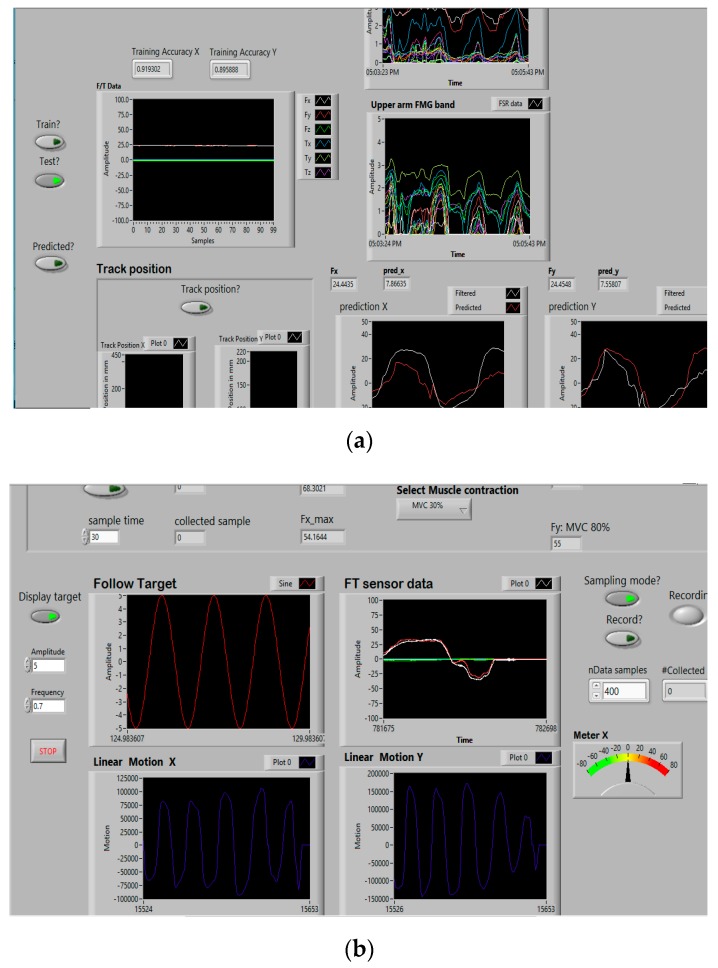
Labview interface of the RT FMG-based integrated controller: (**a**) control pane of FMG-based force estimation, (**b**) display pane of a target motion pattern to follow and maintain muscle volumetric contraction (MVC).

The real-time FMG-based integrated controller was implemented as a Labview interface (Labview 2014, National Instrument, Austin, Texas, USA) that controlled the mechanical system components. It used Matlab scripts (Matlab, Mathworks, Natick, MA, USA) to implement regression models for force estimation with FMG signals, as shown in Figure 4.

### 2.3. Regression Methods

Two supervised regression models, the SVR and the KRR were used for force estimation to control the biaxial stage during interactions with the captured labeled raw FMG signals. These two models were chosen among several other machine learning techniques (such as the multidimensional SVR (MSVR) and the general regression neural network (GRNN)). The selected features and hyperparameters were chosen carefully to create relevant separation among hyperplanes such as L_2_ regularizers, and penalty functions (Cost and Lambda). Data preprocessing, i.e., scaling both training and test datasets (between 0 and 1), was required. The enhanced performances of the selected radial basis function (RBF) kernel features (also known as Gaussian kernel) of both algorithms were observed. The regressors differed in the loss functions, as the SVR used an epsilon-insensitive function and the KRR used a least squared error function. The SVR is a well-known regressor useful for the regression of real-time signals, while the KRR is reported to perform better with small datasets [37]. The selected hyperparameter values used in the study are shown in Table 2. The best values for Cost (c) and Gamma (g) of the SVR model were obtained by grid searches. The regularization penalty (lambda) and the kernel width parameter of the KRR were selected through the parameter trial and error. A single model would be trained either for the x- or y-direction or for both directions, depending on intended arm motions (1-dimentional (1D) or 2D plane). These algorithms were preferred because of lower computational time, special features of the SVR and the KRR (such as a higher number of support vectors and kernel trick), and ability to handle real instantaneous data. Therefore, real-time training and testing of force estimations in motions were possible with their appropriate features and provided reasonably higher performances.

### 2.4. Dynamic Arm Motion Patterns

Five different dynamic arm motions (denoted as M_1_, …, M_5_), i.e., “x-direction (X)”, “y-direction (Y)”, “diagonal (DG)”, “square (SQ)”, and “diamond (DM)” motions, in the manipulator’s cartesian space were considered as intended path trajectories for a participant to interact. These arm trajectory paths included both simple and complex arm movements and were chosen to cover the workspace of the biaxial stage. During interactions, a participant always kept his/her hand grasping the gripper with his/her elbow parallel to the horizonal plane with no specific instructions of grasp moment production. These interactions had 1-DoF and 2-DoF motions, such that the elbow and shoulder rotations were confined in the horizontal plane. The arm movements required shoulder abduction/adduction and elbow flexion/extension while his/her hand grasped the gripper, such that the wrist joint torque direction was in coincidence with the elbow joint torque. Each arm trajectory required unique combinations of elbow and shoulder movements and had spatial-temporal effects vary continuously with a changed direction of motion. For 2-DoF motions, more muscle contractions and expansions happened, while arm movements became progressively frequent, as described below. For data collection and evaluation, all arm motions were performed continuously for a certain time in a sinusoidal motion on the planar surface, with directions as indicated with arrows in Table 3.

#### 2.4.1. Intended 1-DoF Arm Motion Patterns

For X motions, the participant grabbed the gripper/knob mounted on top of linear stage 1 and exerted a hand force in the X-axis only. For Y motions, the gripper was placed on top of the linear stage 2, and the participant grabbed the gripper and then an exerted hand force in the y-axis only. This intended interaction motion pattern required changing the hardware setup and dismounting linear stage 1 in the x-axis.

#### 2.4.2. Intended 2-DoF Arm Motion Patterns

For DG, SQ, and DM interactive arm motions, the participant grasped the gripper and applied a hand force in one of these arm trajectories (Table 3). The applied force in a motion allowed the biaxial stage to adjust its velocity, and the gripper would slide in the same path in the XY planar space. The interaction would continue anticlockwise for a certain time.

In general, people tend to move their arms similarly for certain tasks to minimize energy expenditure tendency [38,39]. Although participants were instructed to interact in certain motions (the general shape of a path to follow with no restriction), the movements were individual-specific. Each participant manipulated the stage in the same way during a certain task to avoid muscle fatigue and energy expenditure during interactions. Considering a torso as a ground (origin) and a hand being fixed as a rigid body by grasping the gripper, the unilateral arm motion was guided by the intended path the participant would choose from redundant degrees of freedom. The full elbow extension (full arm length: 74 ± 4 cm) was avoided (to prevent elbow singularity) [40] and reached the boundary point in the workspace area (to avoid boundary singularity for the biaxial stage). Otherwise, misalignment might happen, and the biaxial stage would fail to follow a compliant trajectory. It is worthwhile mentioning that the range of arm motions and trajectories performed by the participants were affected by hand forces, motions, and ranges of MVCs to maintain muscle fatigue and sitting positions.

### 2.5. Performance Metrics

The coefficient of determination (R^2^) and the normalized root mean square error (NRMSE) were considered for evaluating regression models with force estimation and corrective measurements, as defined in Table 4. R^2^ was used to measure the goodness of fit of the linear regression model by indicating the proportion of a dependent variable explained by an independent variable. It ranges between 0 and 1, while larger values indicating better fit by the model. The NRMSE is usually used for comparing datasets or models with different measurements, with its lower values indicating lower residual variances. Equations (3) and (4) were used for calculating R^2^ and NRMSE, where *Y_i_*, *Y_est_*. and *Y_m_* were the *i*th input, the estimated value, and the mean value, respectively.

*R^2^ =* Explained variation/total variation *=* (Σ(*Y_i_* − *Y_m_*)^2^ - Σ(*Y_i_* − *Y_est_*_._)^2^)/ Σ(*Y_i_* − *Y_m_*)^2^ (3)

NRMSE *=* √(1/*n*(∑*i*(*Y_est_*_._ − *Y_i_*)^2^)/*Y_m_* (4)

Several statistical tests such as one-way ANOVA with single-factor and multivariate tests and two-way ANOVA with repeated measures were performed (IBM SPSS 2.0, NY, USA). A single-factor ANOVA was used to observe the separability of FMG signals during arm flexions and extensions. A two-way ANOVA with repeated measures [41] was performed to assess if arm motion patterns significantly related to the accuracy of regression methods. Five different arm motion patterns and accuracies of two regression models were considered as the two factors. Mauchly’s sphericity tests were employed, and post-hoc tests were performed. The main effects were studied to observe interactions during the motion patterns and the regression model accuracies. The Holm–Bonferroni method was adopted for post-hoc tests, and corrected *p*-values were calculated. One-way ANOVA [41] with multivariate analysis was done to investigate the relationship of the participant’ ages and the performance accuracy.

### 2.6. Protocol

Ten right-handed healthy participants (denoted as P_1_, …, P_10_) with the mean age of 33 voluntarily took part in this study and gave their informed written consents as approved by the Office of Research Ethics at Simon Fraser University, Canada (approval code: 2018s0244). Table 5 shows the descriptive statistics of the participants’ demographics. Among them, one participant was female, and her data fitted well with others.

All participants interacted with the manipulator in five arm motion patterns separately. For each intended motion, there were a data collection phase, two training phases, and two testing phases, termed as a cycle and was described as Algorithm I in Table 6. Training and testing were performed twice for evaluating the two regression algorithms (SVR and KRR) separately. This process was repeated for all five interactive motions and required for around 1.5 h to complete one cycle. Because of this time-consuming nature of the study, only few arm motions with limited directions were investigated. Intended interactions in a certain motion within participants were randomly chosen with periodical rests and controlled MVCs [42]. During training and test phases, regression algorithms were selected randomly. The randomization of motions and regressors helped to avoid observer-expectancy effects. During a cycle, the FMG bands were never removed, when interactions happened in one intended motion. As FMG signals were transient and nonstationary, removing the band would require data collection and training again, as the positions of the sensors would change.

#### 2.6.1. Data Collection Phase 

**Figure 5:**
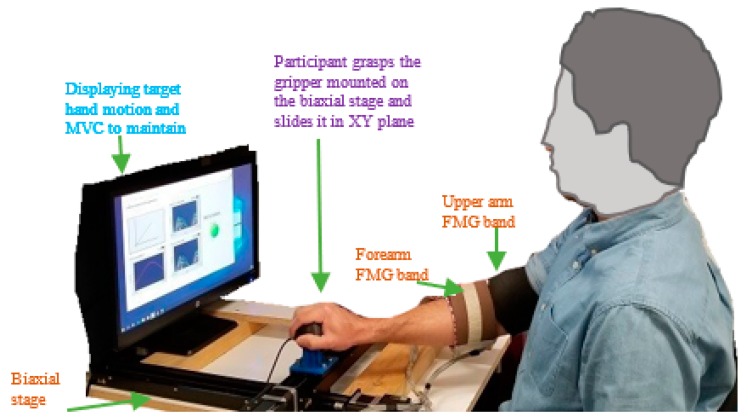
A participant wearing FMG bands interacts with the biaxial stage using an RT FMG-based integrated controller.

At the beginning of the data collection session, the participant sat comfortably on a specialized chair in front of the biaxial stage with his/her shoulder and back straight, while the chair was locked in position, as shown in Figure 5. 

**Figure 6:**
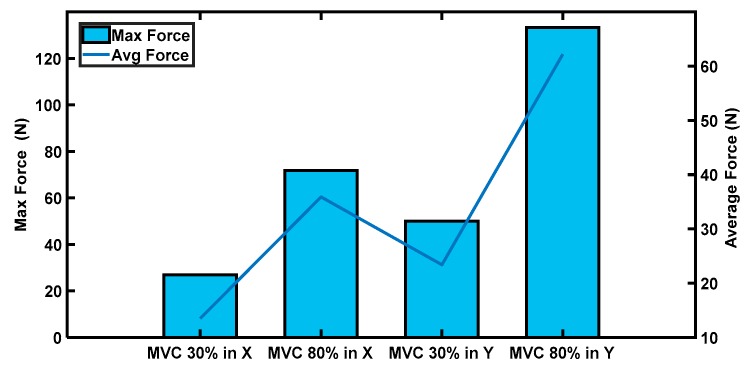
Exerted hand force (the maximum force was what participants could apply; MVCs between 30% and 80% of the average force was exerted by participants (denoted as P_1_, …, P_10_) in interactions).

Two custom designed FMG bands were placed on the forearm and upper arm positions of the participant’s dominant right hand. The data collection phase started by measuring the maximum force that the participant could exert during an intended arm motion for 20 s. Based on this measurement, visual feedback alerted the participant to maintain MVCs above 30% and below 80% for both the x- and y-directions, as shown in Figure 6. 

The controller also provided visual feedbacks displaying target arm motion patterns to follow. Next, the participant exerted forces to the gripper to manipulate the biaxial stage in an intended motion pattern. This was repeated 5 times, while for each repetition 400 records (1 record: 1 × 32 FSR channels and 1 × *F_x_* or *F_y_* in 1-DoF or both in 2-DoF of the label data) of raw data were collected without any filtering. Periodical rests between repetitions allowed the participants to comfort and relax their muscles. For each motion pattern, 2000 records of training data were collected and saved in comma-separated values (csv) files. In this phase, the biaxial stage was controlled by readings from the force sensor. This phase required around 15 min to complete (with periodic rests between repetitions), although the actual data collection duration was less than 10 min.

#### 2.6.2. Training Phase

Once the data collection was done, the next step was to train models using the regression algorithms. The force sensor data were used as true labels for FMG-based force mapping. The collected FMG data were normalized and preprocessed before training using the min-max scaling method. For 1-DoF movements, each regressor generated one trained model (either model x or model y) to estimate hand forces in one direction of an arm motion (X/Y). While for 2-DoF movements (DG/SQ/DM), each regressor generated two trained models for the x- and y-directions (both model x and model y) to estimate forces simultaneously. Models were trained online by calling Matlab scripts in the Labview interface while the participant relaxed, and all trained models were saved; this usually required 2–3 min. Training accuracies and errors were displayed on the Labview interface (Figure 4). All trained models (including the min-max values) were saved as .mat files for the next step of the test phase.

The training process was conducted separately for both regressors and were selected randomly. Offline five-fold leave-one-out cross-validations (LOOCVs) were carried out later for comparing real-time test accuracies.

#### 2.6.3. Test Phase

**Figure 7:**
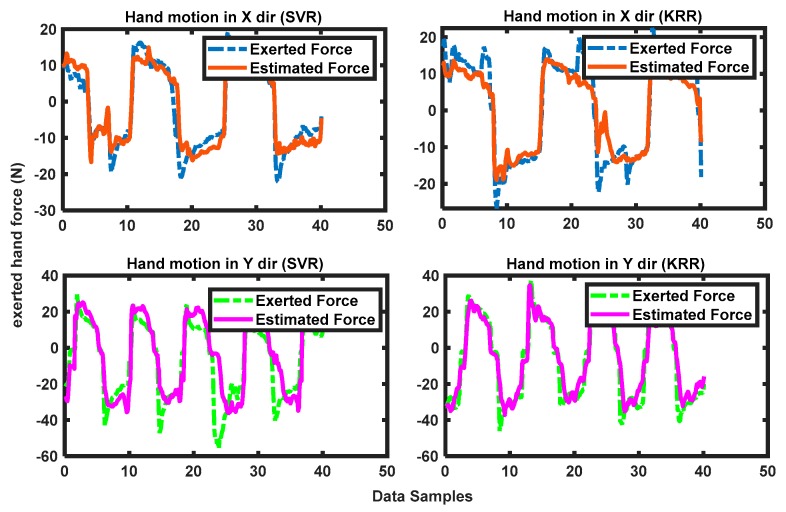
RT test phase, where a participant interacted with the biaxial stage by FMG-based estimated hand forces in intended X and Y arm motions with FMG signals.

The test phase was followed immediately after training; and the trained models were evaluated to estimate forces in real time. Instantaneous test data (FMG data) arriving at the Labview interface were normalized with the scaling values used in the training phase and sent to the regressor, and an exerted hand force was estimated in a dynamic arm motion. The same collaborative task was performed as before, i.e., participants manipulated the stage by grasping its gripper to slide it on the planar surface with an FMG-based estimated force in a certain arm motion and the biaxial stage followed the trajectory immediately. This phase lasted around 2 min, and 1000 records of the test data (labeled FMG signals and estimated hand forces) were collected. The test phase was conducted separately for both regressors. Figure 7 shows the actual exerted forces (true label generator from a force sensor reading) and the estimated force with FMG signals, while a participant was interacting with the stage in X and Y motions in real time with both regressors separately. These plots of real-time test evaluations (true vs. estimated forces) were visible in the control pane of the Labview interface (Figure 4a). Table 7 shows the training data used by the two regressors and the test data (estimated force) collected during the real-time evaluation of each of them.

## 3. Results 

**Figure 8:**
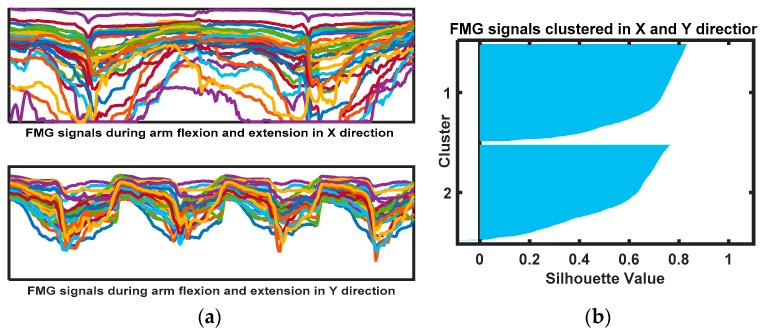
(**a**) FMG signals of arm motions in the x- and y-directions; (**b**) K-means clustering of FMG signals.

Initially, FMG signals were studied to evaluate separability of arm motion directions in a planar space. These signals were collected during one participant performing isotonic muscle contraction of arm flexions and extensions for a certain time in the x- and y-directions, as shown in Figure 8a. The FMG signals (forearm and upper-arm) were fed to the K-means clustering algorithm with a shilloute value of 1, resulting in the clustered FMG signals in the x-direction and the y-direction, as shown in Figure 8b. These FMG signals during flexion and extension (in x- and y- directions) were found statistically significant (*p* = 7.89 × 10^–51^). More specifically, this result meant that FMG signals in x- and y-axes corresponding to arm flexion and extension were distinguishable and revealed the potential of using FMG signals in recognizing arm motion patterns in a planar surface.

FMG-based force estimations in 1D interactions and 2D interactions are described in the following subsections, where cross-validation (CV), training, and test accuracies are presented as box plots of median values.

### 3.1. Real-Time FMG-Based 1-DoF Interactions 

**Figure 9:**
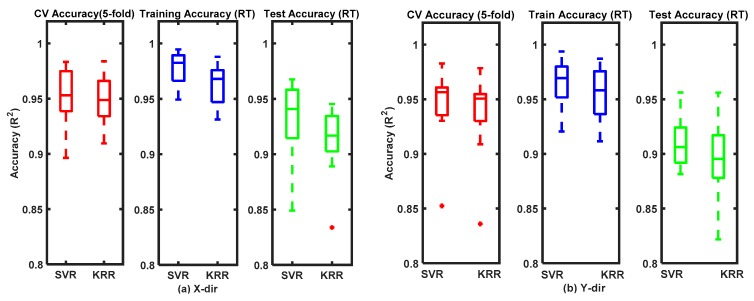
Performances of regressors estimating FMG-based hand forces during intended one-degree-of freedom (1-DoF) arm motions: (**a**) x-direction only; and (**b**) y-direction only.

Figure 9a shows the performance accuracies of the regressors in estimating hand forces in intended X interactive motions with FMG signals. The real-time test accuracies were around 94% and 92% for the SVR and the KRR, respectively, which were comparable with the cross-validation accuracies (around 95%). In intended Y motions, the cross-validation accuracies of hand force estimations were also above 95% for both regressors, although in real time the test accuracies were around 91% and ~90% for the SVR and the KRR, respectively, as shown in Figure 9b. For both intended arm motions, the regressors obtained training accuracies higher than 95%.

### 3.2. Real-Time FMG-Based 2-DoF Interactions 

**Figure 10:**
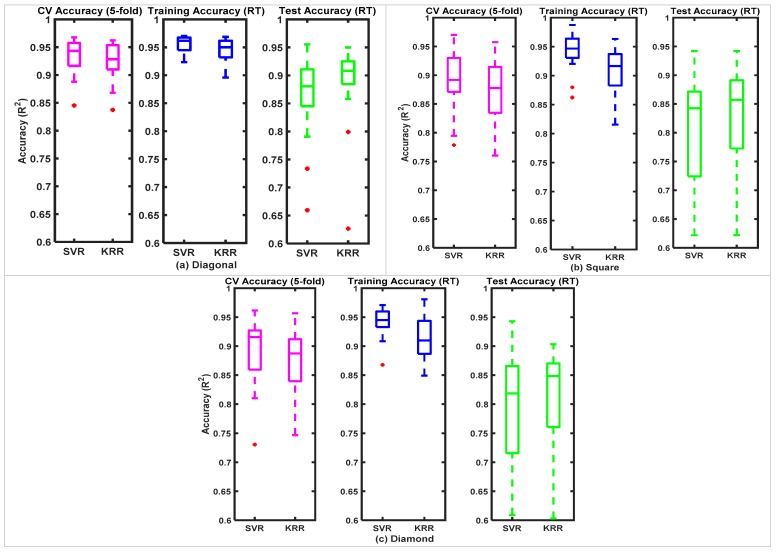
Performances of regressors estimating FMG-based hand forces during intended two-degree-of-freedom (2-DoF) arm motions: (**a**) diagonal; (**b**) square; and (**c**) diamond.

Performance evaluations of the regressors in estimating hand forces during three different arm motion patterns (DG, SQ, and DM) for the participants (denoted as P_1_, …, P_10_) are reported as box plots in Figure 10a–c, respectively, with training accuracies for all patterns higher than 90%. The accuracies of the cross-validations for the DG arm motions pattern were around 94% for both regressors, while the real-time test accuracies were 88% and 91% for the SVR and the KRR, respectively. For the SQ patterns, the accuracies of the cross-validations were 87–89%, while in real-time tests the accuracies were 84% and 86% secured by the SVR and the KRR, respectively. For the DM patterns, the real-time test accuracies of the SVR and the KRR were 82% and 85%, respectively; the cross-validation accuracies were approximately 88–92% for both the regressors.

Performance evaluation of the regressors during force estimation in the real-time test phase in terms of R^2^ and NRMSE (calculated using Equations (1) and (2)) are reported in Table 8 as median values (rounded to ceiling values), which were reasonably notable with higher accuracies and lower errors.

### 3.3. Comparison of Force Estimations in Dynamic Motions 

**Figure 11:**
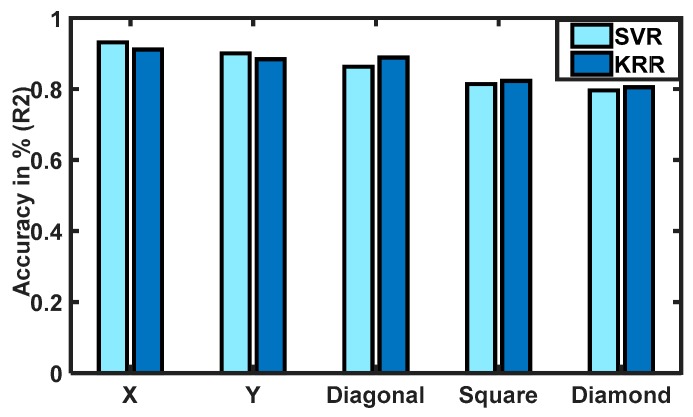
Performances (average R^2^) of the SVR and the KRR in estimating exerted forces with FMG signals during different arm motions.

Figure 11 shows the mean distributions of the test accuracies of the regression models in force estimations for five different motions. Both regressors (SVR and KRR) provided better estimation in 1-DoF arm motions (R^2^ ≥ 90%) than 2-DoF arm motions (R^2^ ≥ 82%). During the 1-DoF interactions, the SVR outperformed the KRR because of model simplicity. While for the 2-DoF interactions, the force estimations in the x- and y-directions were required simultaneously. This required high computational power, considerable memory allocation, and fast communication between the integrated controller and the biaxial stage. Moreover, lower accuracies obtained in the 2-DoF patterns compared to in the 1-DoF arm motions might be due to the increased level of the elbow and shoulder rotations resulting in faster muscle fatigue. For the 2-DoF interactions, the KRR slightly outperformed the SVR in DG and SQ trajectories, although the results were quite comparable. While the SVR required considering all the support vectors of the trained models to estimate, the KRR could provide better prediction with limited samples available in real-time force estimation [36]. Among the 2-DoF arm motion patterns, both the KRR and the SVR performed better in DG patterns because of simpler arm motions and fewer muscle contractions/expansions than in the other two patterns. Although the regressors had lower accuracies in the DM patterns compared to in the other two patterns, real-time force control was achievable with satisfactory accuracies.

With different demographic data, each participant manipulated the stage at his/her own comfortability during an intended motion. For a real-time environment, the regressors had estimated forces that varied among 1-DoF and 2-DoF motions (R^2^ = 82–94%). Studies have shown that changing arm positions adversely influenced the performances of the regression algorithms over the population [43,44], as observed in this study.

The mean absolute error (MAE) shown in Table 9 was also used to compare the regression algorithms’ accuracies in force estimations. The lower the MAE was, the more accurate the regression model was. The MAEs of the SVR were higher than those of the KRR for the DG and SQ patterns, but that of the SVR was lower for the DM patterns compared with that of the KRR. The results indicated that the KRR and the SVR were comparable in the 2-DoF motions while the SVR slightly outperformed the KRR in the 1-DoF motions.

**Figure 12:**
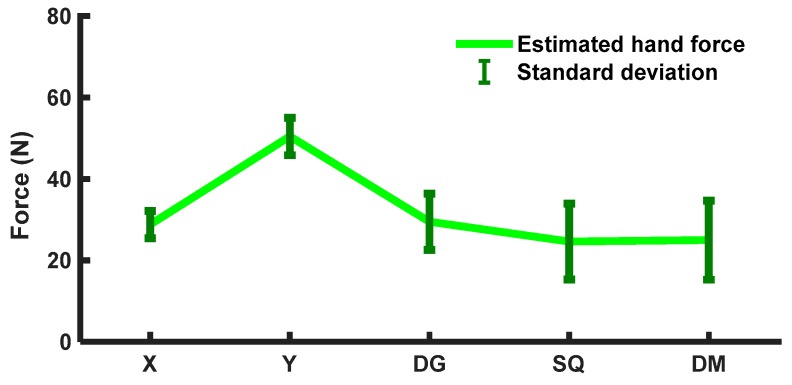
Averaged estimated hand forces and standard deviations (SDs) (within participants) in intended arm motions [X, Y, diagonal (DG), square (SQ), and diamond (DM) patterns].

Figure 12 shows the observed average force estimations with standard deviations (SDs) using FMG signals in different arm motions from participants. Participants applied hand forces (MVC between 30% and 80%), such that the estimated force range was between 20 and 60 N. Demographic data such as height, arm length, arm perimeters, muscle contractions, and interactions varied in wide ranges among participants. For the 1-DoF interactions, less change of directions introduced fewer variations in DoF in arm motions and hence smaller SDs. However, for the 2-DoF interactions, especially in square and diamond motion patterns, more frequent changes in arm directions in the xy-plane were required. This led to more variations among the participants to interact in one motion pattern with their redundant choices of DoF. Thus, variations in SD were higher for the 2-DoF interactions.

The performances of the regressors in estimating hand forces in the 1-DoF interactions were similar (R^2^ ≤ 94% in the x-direction, R^2^ ≤ 91% in the y-direction, as reported in Table 8), and the one-way ANOVA showed no statistical significance among these motions (F = 2.95, p = 1.03). However, participants exerted more hand forces (average estimated F_Y_ ≈ 49 N) to push and pull during Y motions than during any other arm motion interactions, as shown in Figure 12. In both 1-DoF x- and y-directions, arm motions required joint torque combinations of elbow extensions/flexions and shoulder abductions/adductions. In the x-direction, elbow–shoulder combinations were elbow extension ad shoulder extension/horizontal abduction (EE) in the positive x-axis and elbow flexion and shoulder flexion, and horizontal adduction (FF) in the negative x-axis. While applying forces in the y-axis required torques in opposite directions at the shoulder and elbow, such as elbow flexion, shoulder extension, and horizontal abduction (FE) in the positive y-axis and elbow extension and shoulder flexion/horizontal adduction (EF) in the negative y-axis. This contrasted with the fact that human participants could easily interact with the biaxial stage in the horizontal direction (with less exerted force) and required more efforts in the vertical direction of a planar surface (more exerted force). These effects were found similar to those in studies monitoring moments and forces generated by human arms [35,36,45].

### 3.4. Significance in Estimations

An investigation was done to study main effects if there was a significant relation between the arm motion patterns and the accuracies of the regressors. The within-subject-effects tests for arm motion patterns showed statistical significance in different patterns (*F*(4,36) = 9.681, *p* = 0.000). However, it was found that there was no significant difference between the regression models (*F*(1,9) = 0.0251, *p* = 0.877), and no significant interactions between the arm motion patterns and the regression models (*F*(4,36) = 1.144, *p* = 0.352). The post-hoc tests showed statistical significance as X arm motions had performance improvements of 10.3% and 12.1% than those of SQ and DM arm motions, respectively. Likewise, the performance of the Y patterns was 7.4% and 9.2% higher than those of SQ and DM motions, respectively. Pair-wise comparisons of X–SQ and X–DM were statistically significant, while those of Y–SQ and Y–DM were marginally significant, as observed from the corrected *p*-values reported in Table 10.

The one-way ANOVA showed that participants’ ages did not significantly affect the performances of the regression models (*F* = 0.578, *p* = 0.875). There was no relation between the FMG-based force estimation and the participant’ age.

## 4. Discussion

Proper placements of the FMG bands to read the maximum useful information of muscle contractions (elbow and shoulder flexion/extension/adduction/abduction) were selected in accordance with previous studies [46]. For the 2-DoF planar workspace, interactions with the biaxial stage required participants with a combination of elbow and shoulder rotations in the planar space. Positioning the bands in upper-arm (biceps, triceps brachii muscles) and forearm (brachioradialis and extensor carpi radialis muscles) positions, muscle movements were better read. It was observed that using either one of the bands was not enough for force estimation in motions (efficiencies dropped to R^2^ ≤ 45%). The unique characteristics of each arm motion when grasping a force was better captured with more multichannel FMG signals. To recognize versatile complex arm motions in the experimental setup, the 32-multichannel FMG bands provided better estimations.

**Figure 13:**
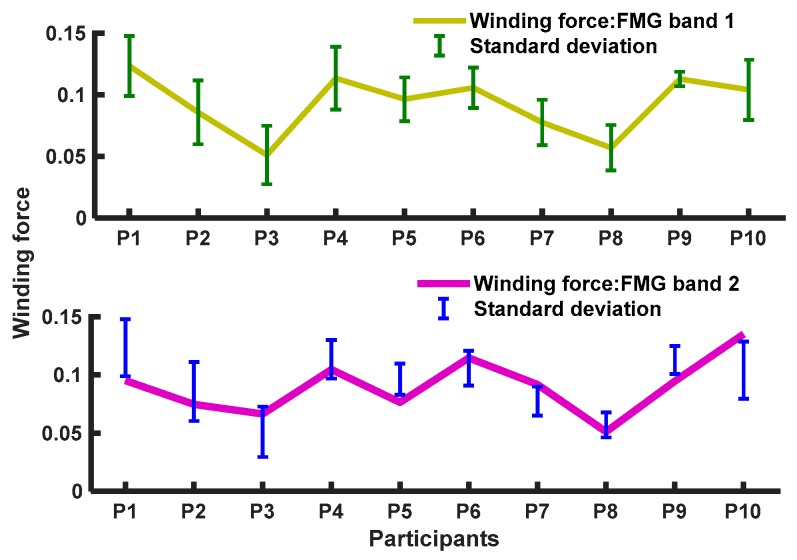
Winding forces of the FMG bands at the beginning of an interaction within participants.

Both bands were placed approximately at the same locations across participants and wrapped on arms moderately to avoid producing some constant pressures on the sensors. Figure 13 shows the winding forces of the bands wrapped on the forearm and upper arm of the participants at an initial time (t = 0-100 ms) waiting to interact (the arm was at rest, while the hand grasped the knob) with individual SDs among participants for each band.

Group differences between the SDs of the two bands’ winding forces were not found statistically significant (*F* = 4.49, *p* = 0. 987), meaning no difference in winding forces were present. As the reported results of the estimated forces in one motion were different than those in the other twos motions (Figure 9 and Figure 10), and the SDs varied significantly among the motions (Figure 12). It could be conclusive that the winding forces (mean ≤ 0.092) did not significantly affect the estimation accuracies of the regressors for individuals due to individual variations in muscle contractions during interactions.

**Figure 14:**
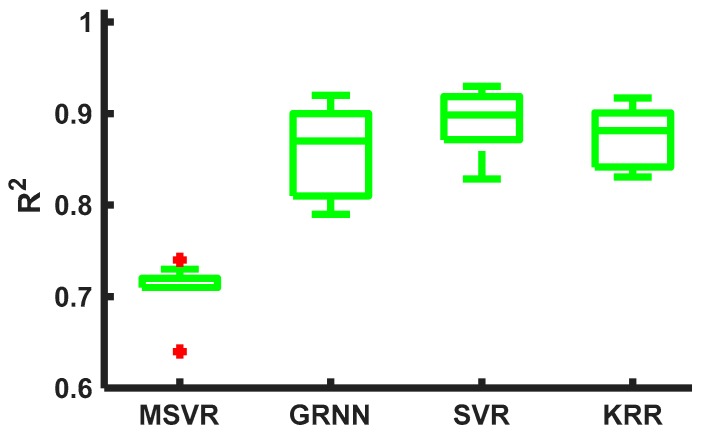
Five-fold cross-validation results for2-DoF interactions in diamond arm motions.

The SVR and the KRR were chosen among several machine learning algorithms and the hyperparameters and features were carefully selected. These algorithms, other than the MSVR (capable of estimating force in one direction while considering forces acting in other dimensions) and the GRNN (a neural network algorithm for regression), were selected for force estimation. The MSVR was appropriate for 2D motions only and did not provide good estimation and therefore was not implemented in real time. Although the GRNN worked well for 1D motions and could achieve comparable cross-validation accuracies to those of the SVR and the KRR, it suffered in real-time 2D interactions (specially in DM motions) because of longer computation time when more complex arm motions are present. Five-fold LOOCVs were carried out for 2D DM motion patterns to justify the selection. Figure 14 shows the force estimation accuracies of these algorithms (for MSVR: R^2^ = 73%, and GRNN: R^2^ = 87%). Among these, the other two algorithms, i.e., SVR and KRR, were implemented in real-time interactions and were proven efficient.

The FMG technique can be a viable alternative to the traditional sEMG. In this study, raw FMG signals were used for learning and estimating, which did not require complex signal preprocessing like sEMG signals [24,25]. A similar study implemented several machine learning classification algorithms to decode discrete hand motion intention using high-density transient EMG signals with a short window (only 150 ms) [47]. A survey on the sEMG technique reported it was adopted in discrete arm motions using classification (approximately 73–98%) and in continuous arm motions and forces using regression (approximately 84–93%) involving offline machine learning and deep learning techniques [48]. Although there is a gap between offline and online performances using sEMG signals, interestingly, the FMG technique was found to perform better in online classification and regression than the sEMG technique [49]. The proposed control scheme in this paper evaluated machine learning techniques in real time, and comparable estimation accuracies (approximately 88–89% averaged across all motions) were obtained with FMG signals, which were in affiliation with the literature. The accuracies of these estimators were higher in 1-DoF in the x-direction, which were 94% and 92% for the SVR and the KRR, respectively; these results were comparable with the reported accuracies of 90% and 92% for the SVR and the KRR, respectively, when one participant interacted with a linear actuator in the x-axis using the FMG technique [50]. The performances of the estimators gradually descended with the increased complexity of 2-DoF arm motion patterns, although being reasonably efficient in real-time interactions.

As a preliminary study, our focus was to investigate the feasibility of FMG-based force estimation in dynamic motions in collaboration with a simple linear robot. The experimental setup was constrained in 2D only, capturing FMG signals were maximized with multidimensional channels, and human interactions were conducted with the biaxial stage with exerted hand forces in dynamic motions. Due to the transient and nonstationary nature of FMG signals and individual-specific muscle contraction during interactive tasks, the changed positions of the FSRs (if the FMG band was removed and put back again) led to retraining the models. The regressors performed well, as the models were trained for estimating forces in a certain motion only; therefore, evaluating all interactions were time-consuming and was not practical. In addition, to capture the unique features when grasping a force in each motion, both bands were required. This might not reflect a practical scenario of HRI, but the outcome proved the viability of the FMG-based control mechanism. The current configuration worked for arm motions considered in two dimensions, which might be different in three-dimensional interactions and require elaborate investigation.

## 5. Conclusions 

Realizing human activities is vital in collaborative tasks, where physical interactions happen between a human worker and a robot. Most common interactions occur by using hands with a contact force and a motion. Estimating hand positions or forces with machine learning techniques are feasible using visual signals but are limited to reliability and range. While biosignals can be wearable and provide flexibility in human movements, it is challenging to intercept human intentions using these signals due to the dynamic nature of interactions. Among many biosignals, the contemporary FMG technique can detect muscle contractions during contact forces. It can be a viable solution to indirect measurements of hand forces, thus understanding human actions. Hence, estimating hand forces in motions in such interactions with FMG signals can be useful for performing collaborative tasks with a robot.

In this paper, a novel scheme of real-time interactions between a human and a biaxial stage via FMG was studied. During interactions, a participant manipulated a stage in a 2D plane with estimated exerted isotonic hand forces to slide a gripper in an intended motion. The biaxial stage would follow the same trajectory by adjusting its velocity, implementing compliant collaboration. The two regression models, SVR and KRR, were implemented for FMG-based force estimation, where accuracies in one dimension, i.e., above 92% in the x-direction and above 89% in the y-direction, were obtained. In 2D complex arm motion patterns such as diamond motions, the estimation accuracies around 82% was obtained; this can be considered as acceptable results in real-time scenarios. Recognizing such arm motion patterns with FMG signals would be beneficial in practical scenarios of collaborative activities. Both regressors were comparable, as no significant differences were found statistical. The probable use of the wearable FMG band by recognizing human activities can enhance pHRI quality in safe collaborations, rehabilitation applications, or prostheses control.

Future studies will be conducted to evaluate the generalization of the FMG-based machine learning model in recognizing unlearned factors in real-time applications during interactions. Implementing a generalized model trained with population data would be able to fit unseen sample data. This would reduce repetitions in training, reduce retaining/recalibration time (if the band is removed and wrapped again) and be applicable for general conditions without compromising estimation accuracies. In addition, optimizing the number of FSRs and combining other sensors such as inertia measurement units (IMUs) in the band could be studied. In future, 3D interactions with FMG signals will be investigated with a 7-DoF robotic arm. Exploring deep learning algorithms (such as convolutional neural networks (CNNs)) might also be useful for learning human intentions using FMG. Exploring advanced algorithms for unsupervised transfer learning would justify the high compatibility of the FMG band for general applications.

## Figures and Tables

**Table 1:** Hardware and devices used.

Devices	
Two force myography (FMG) bands with 16 force-sensing resistors (FSRs) each	▪ Forearm band (500 Hz, 50 samples to read)
	▪ Upper-arm band (500 Hz, 50 samples to read)
Biaxial stage with a gripper/knob (9600 bps)	▪ Linear stage 1 in the x-direction (top)
	▪ Linear stage 2 in the y-direction (bottom) ▪ Gripper as a knob mounted on top of stage 1
Force sensor	▪ Mounted inside the gripper/ knob (500 Hz, 50 samples to read)

**Table 2:** Regression models parameters.

Model	Hyperparameters Ranges	Parameter Selection	Matlab Toolbox
Support vector regressor (SVR)	Cost = 20, Gamma = 1	Grid search	livsvm
Kernel ridge regressor (KRR)	Lambda = 1, Kernel width parameter = 0.9	Trial & Error	Kernel methods toolbox

**Table 3:** Five interactive arm motion patterns.

X	Y	Diagonal	Square	Diamond
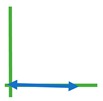	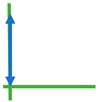	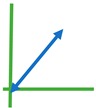	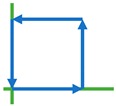	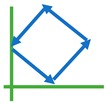

**Table 4:** Performance matrices.

Measures of Estimations		
Accuracy	Coefficient of determination (R^2^)	Equation (3)
Error	Normalized root mean square error (NRMSE)	Equation (4)

**Table 5:** Descriptive statistics of participants.

Feature	Age (year)	Height (cm)	Arm Length (cm)	Upper Arm (cm)	Forearm (cm)
Mean	33	175	74	29	27
Standard deviation	8	5	4	3	3
Mode	35	178	78	29	27

**Table 6:** Algorithm I: Logic flow of RT FMG-based integrated control.

Real-time (RT) FMG-based admittance control of a biaxial stage by an estimated hand force in an intended arm motion pattern	
**Input:** Forearm and upper-arm FMG signals, *x* = [*x_1_*, *x_2_*, …, *x_32_*] True labels from a force sensor, *y* = [*F_x_, F_y_*]	
**Output:** FMG-based estimated force, *y’* = [*F_x_’, F_y_’*] to control the velocity of the biaxial stage	
**Initialization:***z* seconds, *n* data samples, *r* repetitions, *m* reg.model {SVR, KRR}	
1:	**for** *z* **do**
2:	Compute maximum voluntary contraction (MVC) in a planar surface
3:	**end for**
4:	Target_force_H_ ← above MVCs of 30% and below MVCs of 80%
5:	Display ← (Target_force_H_, Intended_motion)
6:	**while** (RT_Data_collection_phase = = true) **do**
7:	*r* = 1;
8:	**repeat**
9:	**for** *n* samples **do**
10:	while (exerted_force = = true) do
11:	Collect *x* and *y* and save them in comma-separated values (csv) format
12:	**end for**
13:	*r* = *r* + 1;
14:	**until** *r* = 5;
15:	**end while**
16:	**while** (RT_Training_phase = = true) **do**
17:	Select *m* reg_model
18:	Select *r* rep csv files
19:	*Trained_model* ← {*x*, *y*}
20:	**end while**
21:	**while** (RT_Test_phase = = true) **do**
22:	Select *m* reg_model
23:	**while** (exerted_force = = true) **do**
24:	FMG-based estimated force, *y′* ← Equation (3)
25:	Velocity of the biaxial stage ← Equation (2)
26:	**end while**
27:	**end while**

**Table 7:** Training data and test data (estimated forces collected during real-time interactions).

Collaborative Task	Training Data (Labeled FMG Signals)	Test Data (Estimated Forces and Labeled FMG Signals)
A participant interacting with the stage by sliding its gripper with an exerted hand force in an intended motion	2000 records or 68,000 data samples	1000 records or 36,000 data samples

**Table 8:** Real-time performance evaluation: R^2^ and NRMSE.

Data Sample	Regression Model	Test Accuracy (R^2^)					Test Error (NRMSE)				
		X	Y	DG *	SQ *	DM *	X	Y	DG *	SQ *	DM *
Training: 2000 records	**SVR**	0.94 ± 0.04	0.91 ± 0.04	0.88 ± 0.07	0.84 ± 0.09	0.82 ± 0.09	0.10 ± 0.05	0.11 ± 0.03	0.10 ± 0.03	0.10 ± 0.04	0.11 ± 0.03
Testing: 1000 records	**KRR**	0.92 ± 0.03	0.90 ± 0.05	0.91 ± 0.07	0.86 ± 0.09	0.85 ± 0.10	0.10 ± 0.04	0.12 ± 0.017	0.09 ± 0.02	0.10 ± 0.04	0.13 ± 0.02

* DG, SQ, and DM stand for diagonal, square and diamond motion patterns.

**Table 9:** Mean absolute errors (MAE) of regression models in different arm motions.

	X	Y	Diagonal	Square	Diamond
**SVR**	0.1091	0.1114	0.1055	0.1092	0.1188
**KRR**	0.1174	0.1154	0.0997	0.1069	0.1262

**Table 10:** Two-way repeated measures ANOVA.

Arm Motion Patterns	Mean Difference	SD	Corrected *p*-Value
X–SQ	0.103	0.013	0.000
X–DM	0.121	0.019	0.000
Y–SQ	0.074	0.022	0.048
Y–DM	0.092	0.026	0.049
